# Antimicrobial Air Filters Using Natural Sea Salt Particles for Deactivating Airborne Bacterial Particles

**DOI:** 10.3390/ijerph17010190

**Published:** 2019-12-27

**Authors:** Sang Bin Jeong, Ki Joon Heo, Byung Uk Lee

**Affiliations:** 1 Graduate School of Energy and Environment, Korea University, Seoul 02841, Korea; sbj315@gmail.com; 2 Aerosol and Bioengineering Laboratory, College of Engineering, Konkuk University, Seoul 05029, Korea; rlwns@konkuk.ac.kr

**Keywords:** bioaerosol, airborne bacteria, filter, natural salt, microbial contaminants, antimicrobial filter

## Abstract

We developed an antimicrobial air filter using natural sea salt (NSS) particles. Airborne NSS particles were produced via an aerosol process and were continuously coated onto the surface of an air filter under various deposition times. The filtration efficiency and bactericidal performance of the NSS-coated filter against aerosolized bacterial particles (*Staphylococcus epidermidis*, *Escherichia coli*) were evaluated quantitatively. The filtration efficiency of the tested filter ranged from 95% to 99% depending on the deposition time, and the bactericidal performance demonstrated efficiencies of more than 98% against both tested bacterial bioaerosols when the NSS deposition ratio was more than 500 μg/cm^2^. The experimental results indicated that the NSS-coated filters have the potential to be used as effective antimicrobial air filters for decreasing environmental exposure to microbial contaminants.

## 1. Introduction

Bioaerosols are defined as aerosols of biological origin, and they include airborne microbial contaminants (e.g., bacteria, fungal spores, and viruses), biological particulate fragments, and a variety of living particles. Bioaerosols can be transported by wind over long distances due to their microscopic size (0.02–100 μm), and some are known to cause human respiratory diseases [1,2,3]. Public concern associated with bioaerosols led to the establishment of legal regulations in healthcare facilities in the Republic of Korea (maximum allowable concentration: 800 colony-forming units (CFU)/m^3^ for total bacterial bioaerosols and 500 CFU/m^3^ for total fungal bioaerosols) [4]. In addition, there are guidelines for bioaerosol concentrations, such as 100 CFU/m^3^ for the standard microbial level of bacterial bioaerosols by the American Conference of Governmental Industrial Hygienists (ACGIH), 300 CFU/m^3^ for fungal bioaerosols by the Indoor Air Quality Association (IAQA), and 150 CFU/m^3^ for total fungal species, which is recommended as a threshold value by the World Health Organization [5,6,7,8]. Currently, controlling bioaerosols is an important issue for protecting humans against airborne pathogens and improving healthcare conditions [9,10].

Among various bioaerosol control methods, such as thermal energy exposure [11,12], ultraviolet germicidal irradiation [13,14], and ion emission [15], filtration technology is the most widely used method to enhance indoor air quality because of its simple installation and low economic costs [16,17]. However, the air filtration method has disadvantages for bioaerosol control. Captured viable bioaerosols on filter surfaces continue to grow by absorbing moisture and nutrients on filter fibers, where they emit foul odors and contaminate the indoor air [3,18]. These viable bioaerosols were also discovered on the surfaces of filtering facepiece respirators [19,20]. The biological properties of bioaerosols, depending on their harmfulness, may have severe effects on human health as compared to non-biological particles. Therefore, the development of an antimicrobial filtration technology that inactivates captured bioaerosols is necessary.

Depositing antimicrobial materials onto filter fibers was suggested as an antimicrobial filtration technology. Recent studies drew attention to antimicrobial air filters using the photocatalytic inactivation method. Various photocatalytic materials, such as TiO_2_ and metal–organic frameworks (MOFs), are being studied for use as antimicrobial air filters [21,22,23]. As such, both inorganic and organic materials were extensively studied as possible materials for antimicrobial filters. In particular, among the various organic materials, natural products were highlighted due to their lower toxicity compared with engineering materials such as silver, copper, and carbon nanotube particles [24,25,26,27]. *Melaleuca alternifolia* (tea tree oil), *Euscaphis japonica*, *Sophora flavescens*, grapefruit seed extract, and propolis were suggested as filter-coating materials to inactivate fungal spores and Gram-positive or -negative bacterial bioaerosols [28,29,30,31]. Specifically, coating filter fibers with submicron-sized antimicrobial particles can enhance antimicrobial activity because of the higher contact surface area.

Natural salt is one of the most popular natural products. It is derived through crystallization of vaporized seawater in a saltern [32] or from the earth’s crust. Natural salt is used as a food preservative, fermentation modifier, toothpaste additive, and antiseptic material since the earliest human societies. Salt particles can inactivate microorganisms by influencing microbial growth parameters, such as osmotic pressure, water molecule movement, temperature, and pH [33,34]. Despite their common usage in human life, the application of natural salt particles in air filtration systems for use against bacterial bioaerosols is yet to be reported.

In this study, we coated air filters with natural sea salt (NSS) particles to examine their antimicrobial performance. We produced submicron-sized salt particles to increase the exposure area to surrounding antigens and observed the physical properties of the NSS particles, such as particle size distribution and morphology. The new air filters with NSS particles were evaluated in terms of filtration efficiency, pressure drop, and antimicrobial characteristics using airborne microorganisms under various particle deposition conditions. The experimental results showed that the NSS air filters have potential as an antibacterial aerosol filtration method for decreasing environmental exposure to microbial contaminants.

## 2. Methods

### 2.1. Preparation of Ethanolic NSS Solution for Aerosol Process

We used NSS particles produced in Korea, which contained NaCl and other plentiful elements, such as Mg, Ca, K, and Zn [35,36]. The salt was pulverized, dissolved in 94% ethanol, and sonicated for 1 h at 40 °C. The solution was passed through 2.5-μm filter paper (Quantitative filter paper, WTM1002-110, Whatman, Maidstone, UK) and a 0.2-μm syringe filter (WTM6784-1302, Whatman, Maidstone, UK) to remove insoluble residue. Finally, a 1% (*w*/*v*) test solution was prepared and stored at 5 °C.

### 2.2. Preparation of Antimicrobial Air Filters

**Figure 1:**
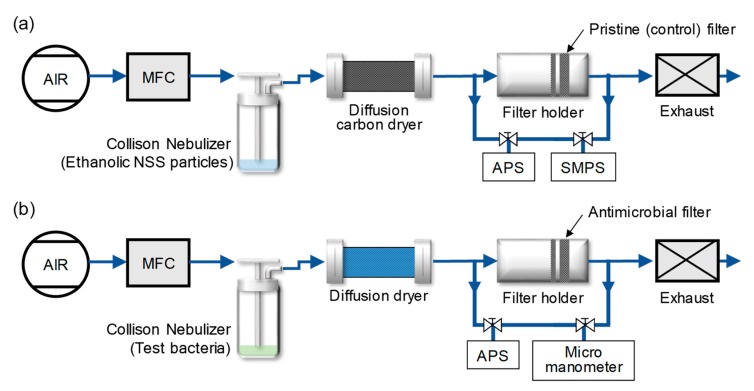
Experimental configuration of (**a**) the antimicrobial filter preparation system, and (**b**) the filtration test system.

Figure 1a illustrates the experimental set-up for the fabrication of filters coated by NSS particles. In the aerosol deposition process, 20 mL of the ethanolic NSS solution was loaded into a six-jet Collison nebulizer (BGI Inc., Waltham, MA, USA). An airflow of 5 L/min, which was cleaned through a high-efficiency particulate air (HEPA) filter, was supplied to the nebulizer. The aerosolized NSS particles were passed through a carbon diffusion dryer to remove any remaining ethanol and continuously deposited onto the polyurethane resin fiber filters. The pristine (control) filter fibers had diameters of 1.02–8.03 μm, a thickness of 0.3 mm, and a packing density of ~33%. The filters were loaded in a filter holder under deposition times of 3, 6, and 9 min. The size distribution and concentration of the aerosolized NSS particles were measured by an aerodynamic particle sizer (APS model 3321, TSI Inc., Shoreview, MN, USA) and a scanning mobility particle sizer (SMPS, model 3080, TSI Inc., Shoreview, MN, USA). The flow rate of the filtered air was adjusted by mass flow controllers (MFC; GMC1200, Atovac, Yong In, Korea).

### 2.3. Preparation of Tested Bacterial Bioaerosols

*Escherichia coli* (Korean Collection for Type Cultures, KCTC 1039, Biological Resource Center, Korea) and *Staphylococcus epidermidis* (KCTC 1917) were selected as antimicrobial test bacteria. *E. coli*, a Gram-negative bacterium, is commonly used in bioaerosol research with *S. epidermidis*, which is Gram-positive [37,38,39]. The bacteria cultures of *E. coli* and *S. epidermidis* were incubated in a nutrient broth medium (beef extract 0.3% and peptone 0.5%; Becton Dickinson, Franklin Lakes, NJ, USA) at 37 °C for 24 h. Stationary-phase organisms were harvested by centrifugation (5000× *g*, 10 min) and washed three times with sterile distilled water. The concentrations of the tested bacterial suspensions were ~10^7^ colony-forming units (CFU)/mL.

### 2.4. Filtration Tests

Figure 1b shows a schematic diagram of the experimental set-up for filtration tests. For generating bacterial bioaerosols, test bacterial pellets were carefully washed three times with sterile distilled water. The bacterial suspension (20 mL) was placed in a six-jet Collision nebulizer, and the bacterial droplets were aerosolized with an airflow of 5 L/min at a pressure of 1 PSIG. The dispersed bacterial droplets were passed through a diffusion dryer to remove moisture, and the dried bioaerosols were introduced onto the surface of the filter medium. The size distributions and concentrations of the bacterial bioaerosols were measured with an aerodynamic particle sizer (APS) at both the inlet and outlet of the filter holder.

The particle filtration efficiency (η) is defined as

η = 1 − (C_outlet_/C_inlet_), (1)

where C_outlet_ and C_inlet_ are the particle concentrations (particles/cm^3^_air_) of the bacterial bioaerosols at the outlet and inlet of the filter holder, respectively. The pressure drop of the filters was measured using a micro-manometer (FC012; Furness Control, Ltd., Bexhill, UK) installed on the inlet and outlet sides of the filter holder. The air face velocity, defined as the average velocity of the air across the face area of the filter, was calculated by dividing the air volume by the total area of the filter. The pressure drop through the NSS-deposited filters was measured using a constant face air velocity.

### 2.5. Antimicrobial Tests

The bactericidal performance of NSS-deposited filters was tested using a culture-colony counting method using bacterial bioaerosols [30]. The bacterial particles were aerosolized at 5 L/min by airstream and deposited onto the tested filters. Both control and antimicrobial air filters were exposed to bioaerosols for 10 min and then placed in 5 mL (V_extraction_) of phosphate-buffered saline (PBS; pH 7.4) containing 0.01% Tween-80. The solutions with these filters were sonicated at 40 Hz for 5 min. Next, the suspensions from the solutions were sequentially diluted and incubated at 37 °C for 18–24 h on nutrient agar plates (beef extract 0.3%, peptone 0.5%, and agar 15%, Becton Dickinson, Franklin Lakes, NJ, USA). After incubation, the colonies on the plates were enumerated and analyzed. We defined the bactericidal performance using the following equations:

BSCF = CFUC_control_/N_control_. (2)

BSAF = CFUC_antimicrobial_/N_antimicrobial_. (3)

N_control_ or N_antimicrobial_ = C_inlet_ × Q_sampling_× η × β_extraction_/V_extraction_. (4)

Bactericidal performance (%) = (1 − BSAF/BSCF) × 100.(5)

In the equations, BSCF is the bacterial survival rate from the control filter, and BSAF is the bacterial survival rate from the antimicrobial filter; N_control_ and N_antimicrobial_ are the concentrations (CFU/mL_suspension_) of bacterial particles in the extraction suspension from the control and the antimicrobial filter, respectively; CFUC_control_ and CFUC_antimicrobial_ are the concentrations (CFU/mL_suspension_) of the culture colonies from bacterial suspensions produced from the control filter and the antimicrobial filter, respectively. C_inlet_ is the bacteria particle concentration at the inlet of the filter holder, Q_sampling_ is the sampled airflow volume, η is the physical filtration efficiency, β_extraction_ is the extraction efficiency from the filters to suspensions, and V_extraction_ is the volume of the extraction suspension [40]. Lastly, the antimicrobial activity, termed as bactericidal performance, was evaluated by the amount by which the survival rate decreased due to NSS particles on the filters. In addition, the physical extraction efficiency of the bacteria-deposited filters was assumed to be identical for all tested filters. Each experiment was performed in triplicate.

### 2.6. Statistical Analyses

Correlation coefficients, linear regressions, and *t*-tests of experimental results were analyzed using SPSS software (ver. 21; SPSS Inc., Chicago, IL, USA).

## 3. Results and Discussion

### 3.1. Characteristics of Aerosolized NSS Particles

**Figure 2:**
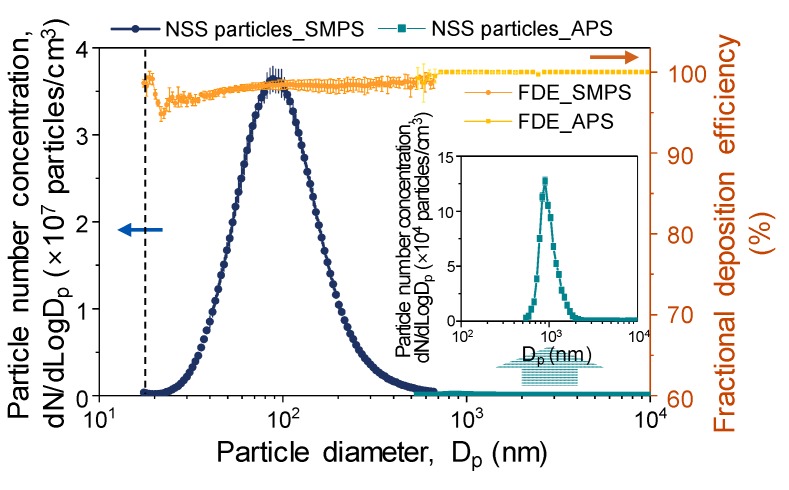
Particle size distribution of natural sea salt (NSS) particles measured by a scanning mobility particle sizer (SMPS) and an aerodynamic particle sizer (APS), with fractional deposition efficiency (FDE) of the control filter. Error bars indicate the standard deviation (*n* = 3).

Figure 2 shows the size distribution of the aerosolized NSS particles measured by APS and SMPS. The particle characteristics were measured by setting the air dilution ratio to 1 (aerosol flow):4 (clean air low), taking into account the measurement limits of the equipment. The nebulization process produced particles with a wide size distribution ranging from several hundred nanometers to 10 μm. The particle size distribution showed a nearly bimodal size distribution of polydisperse curves with peak diameters of 100 nm and 898 nm.

### 3.2. Preparation of Filters Coated by NSS Particles

The fractional deposition efficiencies (FDE) of the generated NSS particles in the filter are also shown in Figure 2. FDE was determined by the particle concentration difference at the filter inlet and outlet, similar to Equation (1). More than 90% of generated particles were deposited on the pristine (control) filters. However, increased deviation of FDE for aerosol particles with diameters ranging from 200 to 600 nm was observed. This relatively fluctuating deviation is attributed to aerosol filtration mechanisms. Aerosols in this range—the most penetrating particles—were too large for diffusion and too small for impaction or interception [2,41]; thus, the deviation of FDE slightly increased in that range.

**Figure 3:**
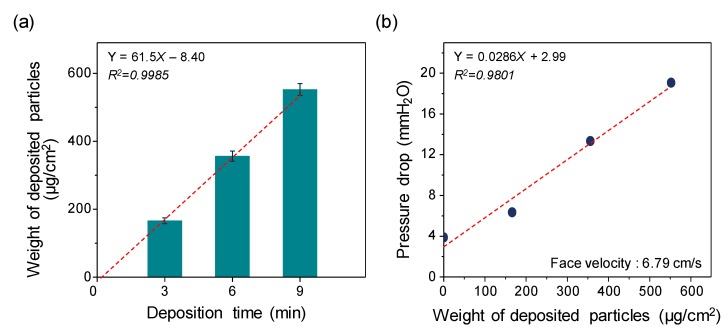
(**a**) The weight of deposited NSS particles under various deposition times, and (**b**) pressure drop through the filters under various deposition weights. Error bars indicate standard deviation (*n* = 3).

**Figure 4:**
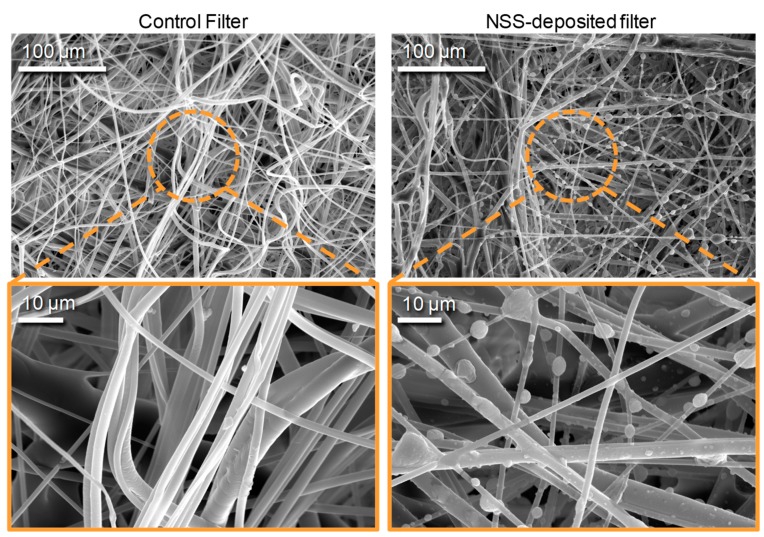
SEM images of the control filters **(left**) and NSS-deposited filters (**right**).

The amount of deposited NSS particles on filters was quantified by comparing the weight of the filters before and after deposition processes using a microbalance (Mettler MT5; Mettler-Toledo International Inc., Korea) with an accuracy of 1 μg (Figure 3a). As the deposition time increased from 3 min to 9 min, the particle deposition weight per cross-sectional filter surface area increased from 166 ± 8.6 to 552 ± 17.5 μg/cm^2^ (*Y* = 61.5*X* − 8.40, *R*^2^ = 0.9985). Figure 3b shows the pressure drop across the filters. The filter pressure drop was proportional to the deposition time of the NSS particles [37]. As the deposition time increased from 0 to 9 min, the filter pressure drop increased from 3.89 ± 0.006 to 19.08 ± 0.024 mm H_2_O at the constant face airflow velocity of 6.79 cm/s (*Y* = 0.0286*X* + 2.99, *R*^2^ = 0.9801). According to Thomas et al. (2001), the deposition of solid aerosol particles can significantly change the pressure drop of fibrous filters on the order of a few grams per square meter of particle deposition [42]. Although the pressure drop of the test filters tended to increase as the NSS deposition rate increased, the difference in the pressure drop among the test filters was statistically insignificant (*p* > 0.05 by a paired *t*-test). Figure 4 shows electron microscope images (200 NANO SEM; FEI Co., Hillsboro, USA) of filters covered by NSS particles. The control filters without NSS particles had smooth fiber surfaces (left image); however, in the filters with NSS particles, the NSS particles were easily observed on the fibers (right image). The particles on the fibers were bumpy and elliptical, and some particles had variegated surfaces (right image).

### 3.3. Filtration Test of NSS-Deposited Filters Using Bacterial Aerosols

**Figure 5:**
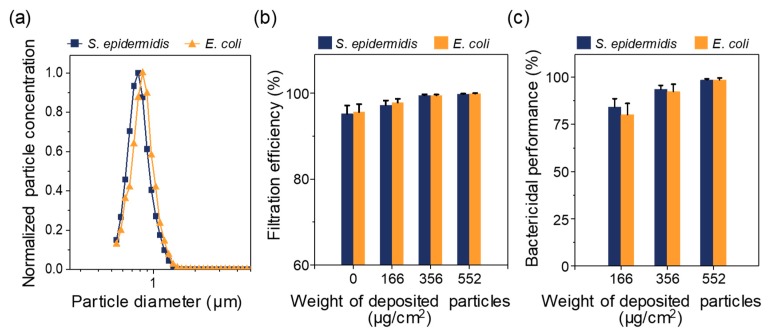
(**a**) The particle size distributions of generated *Staphylococcus epidermidis* and *Escherichia coli* aerosols. (**b**) Filtration efficiencies of the filters with various weights of deposited NSS particles against bioaerosols. (**c**) Bactericidal performance of the filters with various weights of deposited NSS particles against captured bioaerosols. Error bars indicate standard deviations (*n* = 3).

Figure 5a shows the normalized size distributions of *S. epidermidis* and *E. coli* bioaerosols measured by APS. The *S. epidermidis* bioaerosol showed a unimodal curve with a mode (peak) diameter of 0.77 ± 0.01 μm, a geometric mean diameter (GMD) of 0.78 ± 0.014 μm, and a geometric standard deviation (GSD) of 1.22 ± 0.014. The *E.* *coli* bioaerosol also had a unimodal curve, with a mode diameter of 0.84 ± 0.01 μm, a GMD of 0.81 ± 0.007 μm, and a GSD of 1.18 ± 0.011. The number concentrations of aerosolized *S. epidermidis* and *E. coli* bioaerosols were 571 ± 35.2 and 588 ± 49.3 particles/cm^3^_air_, respectively (Table 1).

The aforementioned aerosols were used for evaluating the filtration efficiency of the NSS-deposited filters. The filtration efficiencies of all the tested filters (including the control filter) were greater than 95% against both bioaerosols (Figure 5b). The zero value of the weight of deposited particles refers to the control filter. Because the weight of deposited NSS particles increased, the filtration efficiency tended to increase slightly (~99%) as a result of the aerosol particle deposition. The deposited NSS particles not only filled the porous spaces between the filter fibers but also widened the area where the bacterial particles could be captured, which was also statistically significant (*p* < 0.05). If the deposition rate of NSS particles is increased further, it can be expected that the NSS-deposited filters would exhibit better filtration efficiency. However, increasing the deposition rate also increases the pressure drop of the filters, which causes energy loss. Therefore, the weight of the deposited NSS particles per filter media must be optimized to prevent an excessive increase in the pressure drop.

### 3.4. Antimicrobial Tests of NSS-Deposited Filters Using Bacterial Aerosols

Figure 5c shows the bactericidal performance of NSS-deposited filters against bacterial aerosols. The bactericidal performance was quantified by the bacterial CFU concentration extracted from the control filter, as described in Equations (2)–(5). For *S. epidermidis* bacteria, as the amount of deposited NSS particles increased from 166 to 552 μg/cm^2^, the bactericidal performance increased from 84.2 ± 4.39 to 98.5 ± 0.64%. *E. coli* bioaerosols showed slightly lower efficiencies; the bactericidal performance increased from 80.2 ± 5.92 to 98.6 ± 1.06% under the same weight increase. However, these results were not statistically significant for either bacterial bioaerosol (*p* > 0.05). Gram-negative bacteria have an outer membrane constructed of lipopolysaccharide molecules, which may act as a barrier that increases biochemical resistance [38,43]. Therefore, the antimicrobial effects were slightly higher on *S. epidermidis* when the weight of the deposited NSS particles was low; however, the difference became insignificant as the deposition weight increased.

The antimicrobial effects were derived by osmotic pressure due to the relatively high NSS concentrations, which are widely known to damage proteins [34,44]. Additionally, the dryness of the filter surface due to the NSS particles may have affected bacterial survival. Quan et al. (2017) demonstrated that salt-coated filters are effective in deactivating virus bioaerosols [45]. The biggest difference between the aforementioned research and the current study is the fabrication process of antimicrobial filters. We used the aerosol deposition method, through which the antimicrobial product can be coated onto filter fibers without physical damage. Therefore, higher filtration efficiency and antimicrobial performance at a lower face velocity could be obtained. In addition, antimicrobial air filters using natural products, such as tea tree oil, *Sophora flavescens,* and grapefruit seed extracts show excellent antimicrobial effects on bioaerosols [28,30,31]. However, these products are not easily found in urban living spaces, and additional chemical treatments are required to extract the ingredients. To the best of our knowledge, this is the first reported study of an antimicrobial air filter using natural salt particles against bacterial aerosols. Because the purpose of the research was to reduce bioaerosols in living spaces, there are limitations in studying special species, such as halobacterium. Further studies investigating long-term stability, humidity effects, and effects on viable but non-cultivable (VBNC) bacteria are necessary for applications of these NSS-deposited filters.

## 4. Conclusions

In this study, we evaluated the antimicrobial performance of air filters coated by natural sea salt (NSS) particles against two types of bacterial bioaerosols. Our results indicated that filters with NSS particles inhibited the growth of the two typical indoor environmental bacteria on the air filters (maximum reduction rate: 98%). The bactericidal performance increased with increasing amount of deposited NSS particles. However, the deposited NSS particles increased the pressure drop of the filters. Therefore, the amount of deposited NSS particles needs to be optimized. This study provides practical information regarding the development of a nature-friendly air purification system that can be used in indoor air quality control systems.

## Figures and Tables

**Table 1:** Concentration, peak diameter, geometric mean diameter (GMD), and geometric standard deviation (GSD) of test bacterial bioaerosols (*n* = 3).

Type of Bacteria	Particle Concentration (×10^2^ Particles/cm^3^_air_)	Peak Diameter (μm)	GMD (μm)	GSD
*Staphylococcus epidermidis*	5.71 ± 0.352	0.77 ± 0.01	0.78 ± 0.014	1.22 ± 0.014
*Escherichia coli*	5.88 ± 0.493	0.84 ± 0.01	0.81 ± 0.007	1.18 ± 0.011
